# Supplementary material for: Decoding arm speed during reaching
Source: Nat Commun. 2018 Dec 7;9:5243. doi: 10.1038/s41467-018-07647-3 (PMC6286377; doi:10.1038/s41467-018-07647-3)
Supplement: Supplementary file 4 — Supplementary Information [file 41467_2018_7647_MOESM4_ESM.docx]

# Decoding arm speed during reaching

Inoue et al.

## Supplementary Methods

The basic encoding and decoding equations can be used to derive analytical expressions for the simulation results. These compare decoding performance using firing rates generated by the gain-only or offset models. Since the gain-only model is a simpler version of the offset model, we’ll use the latter as an illustration.

Starting with the offset generative model (c.f. Eq. 4):

$y_{i}\left( t \right)= b_{0i}+b_{xi}v_{x}(t)+b_{yi}v_{y}(t)+b_{si}\left| \mathbf{v}(t) \right|$ (Supplementary Eq. 1)

and the regression model that finds a predicted firing rate, $\hat{y}_{i}\left( t \right)$, given a specified velocity:

$\hat{y}_{i}\left( t \right)=\hat{b}_{0i}+\hat{b}_{xi}v_{x}(t)+\hat{b}_{yi}v_{y}(t)$ (Supplementary Eq. 2)

we can use the regression coefficients, $\hat{b}_{xi}$ and $\hat{b}_{yi}$, to define the population vector:

$\mathbf{pv}(t)=\left[ \hat{v}_{x}(t),\hat{v}_{y}(t) \right]=\left[ \sum_{i=1}^{N} r_{i}\left( t \right)\hat{b}_{xi},\sum_{i=1}^{N} r_{i}\left( t \right)\hat{b}_{yi} \right]$ (Supplementary Eq. 3)

$$r_{i}\left( t \right)=\frac{f_{i}\left( t \right)-\hat{b}_{0i}}{\hat{m}_{i}}$$

$$\hat{m}_{i}=\left| \left[ \hat{b}_{xi}, \hat{b}_{yi} \right] \right|$$

Expanding the *x*-component of the population vector:

$\hat{v}_{x}\left( t \right)=\sum_{i=1}^{N} (b_{xi}\frac{\hat{b}_{xi}}{\hat{m}_{i}})v_{x}(t)+\sum_{i=1}^{N} (b_{yi}\frac{\hat{b}_{xi}}{\hat{m}_{i}})v_{y}(t)+\sum_{i=1}^{N} (b_{si} \frac{\hat{b}_{xi}}{\hat{m}_{i}})\left| \mathbf{v}(t) \right|+\sum_{i=1}^{N} (b_{0i}-\hat{b}_{0i})$ (Supplementary Eq. 4)

The summed constants can be re-labeled:

$$\hat{b}_{\mathrm{xP}}=\sum_{i=1}^{N} (b_{xi}\frac{\hat{b}_{xi}}{\hat{m}_{i}})$$

$$\hat{b}_{\mathrm{yP}}=\sum_{i=1}^{N} (b_{yi}\frac{\hat{b}_{xi}}{\hat{m}_{i}})$$

$$\hat{b}_{\mathrm{sP}}=\sum_{i=1}^{N} (b_{si} \frac{\hat{b}_{xi}}{\hat{m}_{i}})$$

$$\hat{b}_{0P}=\sum_{i=1}^{N} (b_{0i}-\hat{b}_{0i})$$

$\hat{v}_{x}\left( t \right)=\hat{b}_{0P}+\hat{b}_{\mathrm{xP}}v_{x}(t)+\hat{b}_{\mathrm{yP}}v_{y}(t)+\hat{b}_{\mathrm{sP}}\left| \mathbf{v}(t) \right|$ (Supplementary Eq. 5)

$$\hat{\theta}=\mathrm{atan} \left( \frac{\hat{b}_{\mathrm{yP}}}{\hat{b}_{\mathrm{xP}}} \right)=\hat{X}axis$$

$\hat{v}_{x}\left( t \right)=\hat{b}_{0P}+\left| {\hat{\mathbf{b}}}_{P_{x}} \right|\cdot\left| \mathbf{v}(t) \right|\cdot\cos\left( \theta(t)-\hat{\theta} \right)+\hat{b}_{\mathrm{sP}}\left| \mathbf{v}(t) \right|$ (Supplementary Eq. 6)

Note that a different set of coefficients will be found for $\hat{v}_{y}\left( t \right)$ because the $\frac{\hat{b}_{xi}}{\hat{m}_{i}}$ term becomes $\frac{\hat{b}_{yi}}{\hat{m}_{i}}$.

For the gain-only model:

$\hat{v}_{x}\left( t \right)=\hat{b}_{0P}+\left| {\hat{\mathbf{b}}}_{P_{x}} \right|\cdot\left| \mathbf{v}(t) \right|\cdot\cos\left( \theta(t)-\hat{\theta} \right)$ (Supplementary Eq. 7)

For the Direct Regression decoder, the $\hat{b}_{xi}$’s are found through regression:

$$\hat{v}_{x}\left( t \right)=\sum_{i=1}^{N} \hat{b}_{xi}y_{i}(t)$$

Expanding $y_{i}$:

$\hat{v}_{x}\left( t \right)=\sum_{i=1}^{N} \hat{b}_{xi}(b_{0i}+b_{xi}v_{x}+b_{yi}v_{y}+b_{si}\left| \mathbf{v}(t) \right|)$ (Supplementary Eq. 8)

The summed constants can be relabeled:

$$\hat{b}_{\mathrm{xP}}=\sum_{i=1}^{N} ({\hat{b}_{xi}b}_{xi})$$

$$\hat{b}_{\mathrm{yP}}=\sum_{i=1}^{N} (\hat{b}_{xi}b_{yi})$$

$$\hat{b}_{\mathrm{sP}}=\sum_{i=1}^{N} ({\hat{b}_{xi}b}_{si})$$

$$\hat{b}_{0P}=\sum_{i=1}^{N} ({\hat{b}_{xi}b}_{0i})$$

$$\hat{\theta}=\mathrm{atan} \left( \frac{\hat{b}_{\mathrm{yP}}}{\hat{b}_{\mathrm{xP}}} \right)=\hat{X}axis$$

$\hat{v}_{x}\left( t \right)=\hat{b}_{0P}+\left| {\hat{\mathbf{b}}}_{P_{x}} \right|\cdot\left| \mathbf{v}(t) \right|\cdot\cos\left( \theta(t)-\hat{\theta} \right)+\hat{b}_{\mathrm{sP}}\left| \mathbf{v}(t) \right|$ (Supplementary Eq. 9)

Population vectors were constructed from simulations based on the gain-only simulations (Supplementary Eq. 7, Fig. 5e-h), to obtain the following equations:

$\hat{v}_{x}\left( t \right)=-0.003+0.933 \left| \mathbf{v}(t) \right|\cos\left( \theta(t)-(-0.352^{\circ}) \right)$ (Supplementary Eq. 10)

$\hat{v}_{y}\left( t \right)=0.010+0.934 \left| \mathbf{v}(t) \right|\cos\left( \theta(t)-89.9^{\circ} \right)$ (Supplementary Eq. 11)

The fit directions for the *x* and *y* components (-0.352 and 89.9 degrees) match their expected orientation along the *x* and *y* axes. The *x* and *y* gain coefficients (0.933 and 0.934) are equivalent and the overall offset terms ($b_{0P}= -0.003, 0.010$) are small. This shows that neuronal discharge that obeys the gain-only model is accurately decoded, even for a non-uniform sample of preferred directions.

When the same analysis is applied to the rates simulated with the offset model and a non-uniform distribution of preferred directions (Supplementary Eq. 6, Figs. 5m-p), the decoded output is distorted as indicated by the constant terms:

$\hat{v}_{x}\left( t \right)=7.82+0.933 \left| \mathbf{v}(t) \right|\cos\left( \theta-0.558^{\circ} \right)+(-0.809 \left| \mathbf{v}(t) \right|)$ (Supplementary Eq. 12)

$\hat{v}_{y}\left( t \right)=1.90+0.935 \left| \mathbf{v}(t) \right|\cos\left( \theta-90.2^{\circ} \right)+(-0.196 \left| \mathbf{v}(t) \right|)$ (Supplementary Eq. 13)

Again, the *x* and *y* gain coefficients (.933 and .935) are equivalent and the fit directions (0.558 and 90.2 degrees) are accurate. However, the offsets were relatively large ($b_{0P}=7.82, 1.90 b_{\mathrm{sP}}= -0.809, -0.196$) indicating that offsets for this condition and sample of preferred directions do not tend to cancel out when combined. In contrast, the Direct Regression Decoder (Supplementary Eq. 9, Fig. 6, a-d) had offset coefficients that were small ($b_{0P}=0.411, 0.128 b_{\mathrm{sP}}=-0.048, -0.014$) suggesting that this decoder acts to minimize the offset terms.

**Supplementary Table 1** Decoder parameters based on simulated firing rates from a non-uniform distribution of simulated units.

|  | Gain-only | | Offset | | | |
| --- | --- | --- | --- | --- | --- | --- |
|  | Minimal OLE | | Minimal OLE | | Direct Regress | |
| Variable | *X* | *Y* | *X* | *Y* | *X* | *Y* |
| $\left\vert{\hat{\mathbf{b}}}_{P \cdot} \right\vert$ | .933 | .934 | .933 | .935 | .912 | .928 |
| $\hat{\theta}$ | -.352 | 89.9 | .558 | 90.2 | -.902 | 91.6 |
| $\hat{b}_{\mathrm{sP}}$ | - | - | -.809 | -.196 | -.048 | -.014 |
| $\hat{b}_{0P}$ | -.003 | .010 | 7.82 | 1.90 | .411 | .128 |

The simulation was based on a gain-only- (‘Gain-only) or an offset- (‘Offset’) generative model using the minimal OLE decoder. The offset model was also used to generate the firing rates used in the Direct Regression decoder. See Supplemental Methods for detailed derivation of the variables.

**Supplementary Table 2** Closed-loop BCI session parameters.

| Day | Decoder | # of trials per target | $s_{0}$  (cm s^-1^) | Speed gain | Center-hold (ms) | Target-hold (ms) |
| --- | --- | --- | --- | --- | --- | --- |
| 1 | ANN | 50 | 15 | 1.0 | 300-400 | 450-550 |
|  | OLE | 23 | 15 | 1.0 | 250-350 | 400-500 |
| 2 | OLE | 50 | 12 | 1.0 | 200-300 | 400-500 |
|  | ANN | 43 | 15 | 1.0 | 400-500 | 400-500 |
| 3 | Dir Reg | 53 | 15 | 1.0 | 400-500 | 400-500 |
|  | OLE | 36 | 12 | 1.0 | 200-300 | 400-500 |
| 4 | OLE | 45 | 15 | 0.8 | 200-300 | 400-500 |
|  | Dir Reg | 40 | 15 | 1.0 | 400-500 | 400-500 |
| 5 | Dir Reg | 50 | 15 | 1.2 | 400-500 | 400-500 |
|  | ANN | 45 | 15 | 1.0 | 400-500 | 400-500 |
| 6 | ANN | 45 | 15 | 1.0 | 450-550 | 450-550 |
|  | Dir Reg | 38 | 15 | 1.0 | 450-550 | 450-550 |

The OLE decoder used in BCI sessions was the variance-only OLE. The goal was to collect 45-50 trials per target in each session. This was not achieved in some sessions due to loss of motivation, especially for OLE on day 1 when “drifting” severely deteriorated performance. Subsequently, task parameters were modified for OLE to increase success rate and maintain motivation. This includes using a shorter center-hold time, and using either a slower reference speed $s_{0}$ during decoder calibration (day 2 and 3) or a smaller speed gain factor during full brain control (day 4).
