# Supplementary material for: Decoding arm speed during reaching
Source: Nat Commun. 2018 Dec 7;9:5243. doi: 10.1038/s41467-018-07647-3 (PMC6286377; doi:10.1038/s41467-018-07647-3)
Supplement: Supplementary file 6 — Description of Additional Supplementary Files [file 41467_2018_7647_MOESM6_ESM.pdf]

Description of Additional Supplementary Files

File Name: Supplementary Movie 1
Description: Monkey N center-out task using a BCI with the variance-only OLE decoder. This movie was reconstructed using cursor positions recorded during online closed-loop control. The movie shows one block of 11 consecutive trials from day 3, including 8 successful trials (one for each target) and 3 failed trials (one failed center-hold and two failed target-hold). This block is one of those that have fewer failed trials (n<4; 19% of all OLE blocks) and contain both center-hold and target-hold errors. Note that the task was designed to reset the cursor to the center at the start of each trial.

File Name: Supplementary Movie 2
Description: Monkey N center-out task using a BCI with the Direct Regression decoder. All 8 trials (one for each target) in this block of the experiment were successful. This block was randomly chosen from those blocks with no failed trial (19% of all blocks using the Direct Regression decoder).

File Name: Supplementary Movie 3
Description: Monkey N center-out task using a BCI with the ANN decoder. All 8 trials (one for each target) in this block of the experiment were successful. This block was randomly chosen from those blocks with no failed trial (42% of all blocks using the ANN decoder).
